# Trends in global amyotrophic lateral sclerosis research from 2000 to 2022: A bibliometric analysis

**DOI:** 10.3389/fnins.2022.965230

**Published:** 2022-08-10

**Authors:** Guanzhong Shi, Jinxia Zhou, Kun Huang, Fang-Fang Bi

**Affiliations:** ^1^Department of Neurology, Xiangya Hospital, Central South University, Changsha, China; ^2^Xiangya School of Medicine, Central South University, Changsha, China; ^3^National Clinical Research Center for Geriatric Disorders, Xiangya Hospital, Central South University, Changsha, China

**Keywords:** bibliometrics, amyotrophic lateral sclerosis (ALS), research trends, publication characteristics, collaboration

## Abstract

**Background:**

Amyotrophic lateral sclerosis (ALS) is a relentlessly progressive neurodegenerative disease affecting the motor neurons. Although much research has been conducted in this field, few bibliometric studies have been conducted. This study aimed to provide an overview of publishing characteristics and trends in ALS research since 2000 using a bibliometric analysis.

**Methods:**

We conducted a comprehensive literature search in the Web of Science (WOS) Core Collection database for scientific output related to ALS from 2000 to 2022. The retrieved dataset was refined using Google OpenRefine and analyzed using bibliometrix.

**Results:**

A total of 29,391 articles published since 2000 were retrieved, with an average annual growth rate of 6.35%. Ninety-six countries and regions contributed to ALS research, among which the United States had the dominant position with the highest number of publications (*n* = 8,202) and citations (*n* = 558,561). An association analysis was performed to form networks of country collaboration and keyword co-occurrence. The evolution of topic trends was demonstrated in terms of both frequency and proportion.

**Conclusion:**

The output of ALS research has increased steadily over the years, and the United States and Western Europe are leaders in this field. There is an upgradation in the pathomechanism and clinical research on ALS.

## Introduction

Amyotrophic lateral sclerosis (ALS) is a fatal and incurable neurodegenerative disease characterized by degeneration of the motor neurons in the brain and spinal cord, leading to motor and extra-motor symptoms (Brown and Al-Chalabi, 2017). Patients with ALS experience systemic progressive muscle atrophy and eventually die from dyspnea (Brown and Al-Chalabi, 2017). In Europe and the United States, the incidence of ALS is approximately 2.3 per year per 100,000 individuals, while the prevalence varies from 5.2 to 6.2 per 100,000 individuals (Chiò et al., 2013; Xu et al., 2020). ALS is traditionally classified into two categories: familial ALS (fALS), which is caused by mutations in a heterogeneous set of genes, and sporadic ALS. The age of clinical onset of ALS is highly variable, but is almost always after the fourth decade of life. Juvenile ALS is rare and only occasionally seen in patients with fALS (Robberecht and Philips, 2013). However, there is no clear description of the mechanisms underlying ALS.

Bibliometrics uses mathematical and statistical methods to analyze and quantitatively assess the contribution and productivity of a field of study, including countries, journals, and authors (Durieux and Gevenois, 2010). Bibliometric research is used to clearly show the publishing characteristics, keyword associations, and research trends in a particular field. It provides an overview and evolution of knowledge topics that can help guide decision-making (Broadus, 1987).

The first modern medical description of ALS dates back to 1881 when Ferrier published a case report on the symptoms of patients with ALS in *The Lancet* (Ferrier, 1881). Over the past few decades, technological advances in genetics, immunology, molecular biology, and imaging have encouraged researchers worldwide to publish numerous articles to better understand the diagnosis, mechanism, and treatment of ALS. However, there is no bibliometric study on the trends and associations in ALS research.

In this study, we performed a bibliometric analysis of published literature related to ALS in the Web of Science (WOS) since 2000, which, to our knowledge, has not been performed to date. We expect this bibliometric analysis to provide insights into articles on ALS and their authors and associations and trends in ALS research to better understand current research gaps and future research orientations.

## Materials and methods

Data from ALS research conducted from 2000 to 2022 were retrieved from the WOS Core Collection database on May 20, 2022 using the following keywords: “ALS” OR “motor neuron disease” OR “Lou Gehrig’s disease.” The language was restricted to English and the document type was limited to articles and reviews only. As the data were retrieved from a single database, there were no duplicates. The full records and cited references were exported in the format of both plain text (for analysis in bibliometrix) and tab-delimited text (for keyword refinement and manual combination). Keywords in the metadata were retrieved and cleaned using Google OpenRefine, which has several natural language clustering algorithms for clustering keywords with similar spellings. Identical keywords of different forms were detected and combined. Manual combination was also performed to combine keywords that were not similar in spelling, such as “ALS” and “amyotrophic lateral sclerosis.” This step was conducted to ensure the accuracy of keyword counts, associations, and trends.

The plain text data were imported and converted to the dataframe format and merged with the refined tab-delimited file using the bibliometrix package (version 3.2.1) in the R software (version 4.2.0) (Aria and Cuccurullo, 2017). R is an open-source programming language for statistical computing and graphics and provides a wide variety of statistical and graphical techniques. Bibliometrix offers a comprehensive platform for bibliometric analysis, including the calculation of the frequencies of articles, authors, keywords, countries, and citations and building of data matrices for co-citation, scientific collaboration analysis, and co-occurrence analysis. The merged data were analyzed for the output of countries and authors, topic dynamics, and research associations. The dataset statistics were exported and plotted using the ggplot2 package (version 3.3.6), a declarative graphic creation system based on graphic syntax used to create elegant data visualizations. All figures were embellished using Adobe Illustrator.

## Results

As shown in Table 1, 29,391 articles related to ALS conducted from 2000 to 2022 were retrieved from the WOS database. The number of publications in ALS research had increased dramatically from 635 in 2000 to 2,315 in 2021, with an average annual growth rate of 6.35%, suggesting a relatively stable growth trend. Each included manuscript was cited 45.61 times on average, and the annual citation rate was 4.582%, suggesting that the potential for citation was relatively high. The average number of authors of each article was 2.77, and only a few articles (*n* = 1,414) were conducted by single authors, indicating that cooperation is common in ALS investigations.

**TABLE 1:** Main information of the bibliometric dataset.

Dataset main information	
Timespan	2000∼2022
Sources (journals, books, etc.)	3,038
Documents	29,391
Average years from publication	8.54
Average citations per document	45.61
Average citations per year per doc	4.582
References	706,787
**Document types**	
Article	21,699
Article; book chapter	69
Article; data paper	11
Article; early access	185
Article; proceedings paper	719
Article; retracted publication	8
Review	6,515
Review; book chapter	133
Review; early access	52
**Document contents**	
Keywords plus (ID)	31,524
Author’s keywords (DE)	34,276
**Authors**	
Authors	81,330
Authors of single-authored documents	1,056
Authors of multi-authored documents	80,274
**Authors collaboration**	
Single-authored documents	1,414
Documents per author	0.361
Authors per document	2.77
Collaboration index	2.87

### Country analysis

**FIGURE 1:**
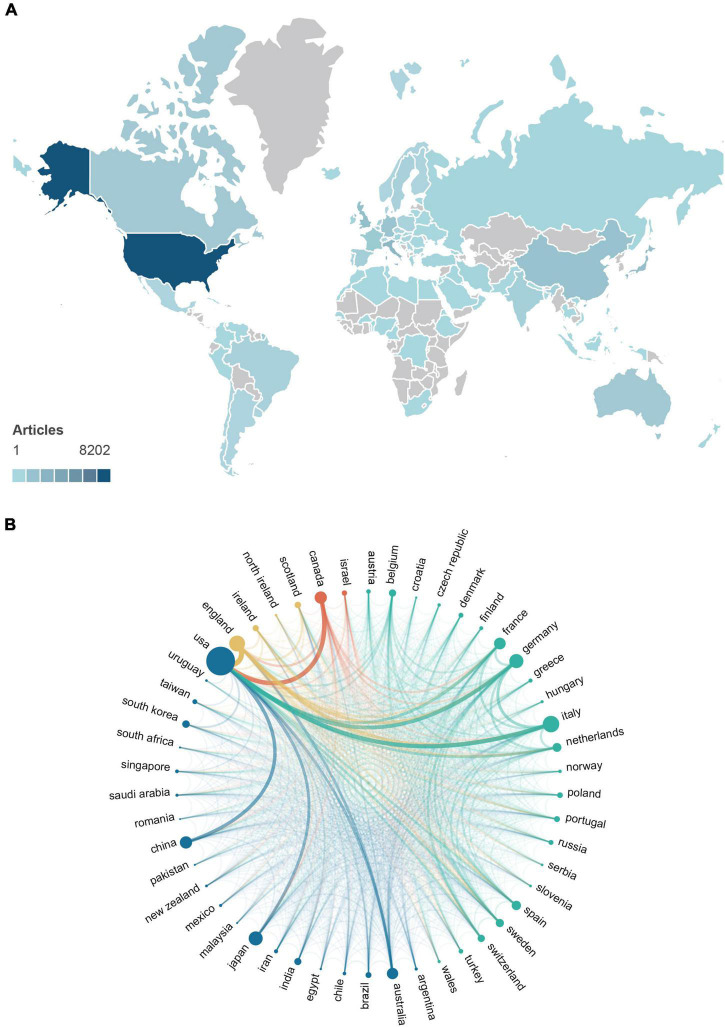
**(A)** Distribution of countries contributing to ALS investigation (the depth of the color represents the number of documents from the country or region; gray represents countries with no publications). **(B)** Collaboration network of the top 50 countries with the highest number of publications (the countries that frequently collaborate are classified in the same color; the circle size indicates the number of publications; and the line thickness indicates the number of co-authored articles).

The global contribution to ALS research was analyzed, and an overview of the top 10 countries in terms of publications is listed in Table 2. We counted all countries contributing to ALS investigations and color-coded their contributions on a world map (Figure 1A). A total of 96 countries and regions contributed to the publications in this field. The United States published the highest number of articles (*n* = 8,202, 28.10%), followed by Italy (*n* = 2,620, 8.96%), England (*n* = 2,166, 7.41%), Japan (*n* = 2,075, 7.10%), China (*n* = 1,767, 6.05%), Germany (*n* = 1,651, 5.65%), Canada (*n* = 1,255, 4.29%), Australia (*n* = 1,240, 4.24%), France (*n* = 1,176, 4.02%), and Spain (*n* = 743, 2.54%).

**TABLE 2:** Overview of top 10 countries with the highest number of publications.

Country	Articles	Frequency	Total citations	Average article citations	SCP	MCP	MCP ratio
United States	8,202	0.281	558,561	68.1	6,570	1,632	0.199
Italy	2,620	0.0896	93,676	35.75	2,019	601	0.229
England	2,166	0.0741	116,017	53.56	1,387	779	0.36
Japan	2,075	0.071	65,250	31.45	1,816	259	0.125
China	1,767	0.0605	36,380	20.59	1,479	288	0.163
Germany	1,651	0.0565	74,318	45.01	1,081	570	0.345
Canada	1,255	0.0429	67,336	53.65	835	420	0.335
Australia	1,240	0.0424	46,968	37.88	900	340	0.274
France	1,176	0.0402	44,747	38.05	816	360	0.306
Spain	743	0.0254	21,256	28.61	563	180	0.242

SCP, single country paper; MCP, multiple country paper.

The articles published in the United States received the most citations (*n* = 558,561), followed by those in England (*n* = 116,017), Italy (*n* = 93,676), Germany (*n* = 74,318), Canada (*n* = 67,336), Japan (*n* = 65,250), Australia (*n* = 46,968), France (*n* = 44,747), China (*n* = 36,380), and the Netherlands (*n* = 24,710). Among the top 10 countries in terms of the number of articles published, the average number of citations of the articles published in the United States and England was 68.1 and 35.75, respectively, while that in China and Spain was relatively lower at 20.59 and 28.61, respectively.

As illustrated in Figure 1B, the cooperation network between countries and regions could be divided into four clusters among the top 50 countries participating in ALS research. The blue cluster was centered in the United States. The core cooperating countries were Australia, China, and Japan, and other countries, including India, Malaysia, and South Korea. The green cluster included Italy, Germany, and France as the axis of cooperation and other European Union countries, such as Switzerland, the Netherlands, and Belgium. The red cluster included Canada and Israel, while the yellow cluster included England, Scotland, Northern Ireland, and Ireland.

### Author analysis

A total of 81,330 authors had contributed to ALS research since 2000. The total number of citations, number of publications, H index, G index, and country of the 10 most cited authors in this field are presented in Table 3. More than half of the authors were from the United States, two from England, one from Canada, and one from Italy. This finding demonstrates the leadership of the United States in ALS research.

**TABLE 3:** Overview of top 10 most influential authors in ALS research.

	Total citations	Number of publications	H index	G index	Country
Trojanowski JQ	30,043	168	86	168	United States
Shaw CE	23,550	198	72	152	England
Al-chalabi A	23,477	244	69	150	England
Brown RH	21,264	171	73	145	United States
Chio A	20,876	266	68	139	Italy
Hardiman O	19,469	285	68	133	England
Van Den Berg LH	18,491	302	72	126	Netherlands
Shaw PJ	16,242	257	69	119	England
Leigh PN	15,162	159	61	121	England
Robberecht W	14,830	153	65	120	Belgium

John Q. Trojanowski was the most cited author with the highest H index in ALS research. As a professor at the Center for Neurodegenerative Disease Research, University of Pennsylvania School of Medicine, John Q. Trojanowski focused on the molecular mechanisms of neuronal dysfunction, degeneration, and death in normal aging and neurodegenerative diseases. His most cited article on ALS, titled “Ubiquitinated TDP-43 in frontotemporal lobar degeneration and ALS,” was published in *Science* in 2006. With 4,034 citations, this article was the most cited research article in the field (Neumann et al., 2006).

**FIGURE 2:**
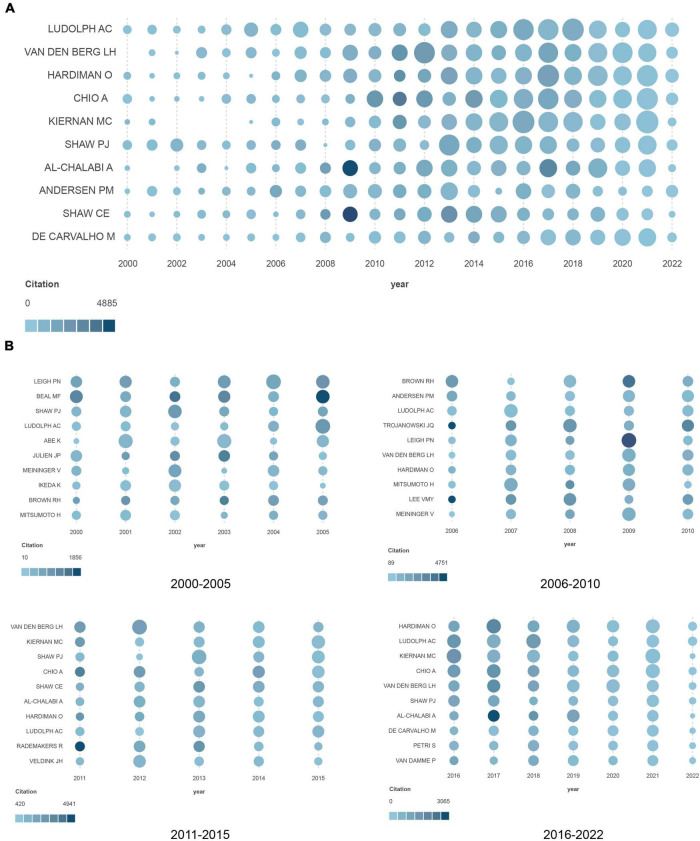
**(A)** Productivity over time of the top 10 most productive authors. **(B)** Output of the 10 most productive authors in different periods (the circle size represents the number of articles published by the authors, while the number of citations per article is expressed by the depth of the color).

As shown in Figure 2A, we calculated the output of the 10 most productive authors over time. The number of articles published by the authors was expressed by the dot size, while the number of citations per article was expressed by the dot color. Notably, the number of publications increased significantly after 2010 compared with that before. However, the year with the highest number of citations remained around 2010. A subgroup analysis by time period on the output of the 10 most productive authors in each period is presented in Figure 2B.

### Most cited documents

Information on the 10 most cited documents is listed in Table 4. Among these documents, seven were reviews, while the remaining three were research articles. The most cited document was that by Finkel and Holbrook (2000), who reviewed evidence that the production of oxidants and the ability of organisms to respond to oxidative stress are closely related to aging. The most cited article was that on TDP-43 by Neumann et al., who demonstrated that hyper-phosphorylated and ubiquitinated TDP-43 is a major component of ubiquitin-positive inclusions in the neurons of patients with frontotemporal dementia and ALS (Neumann et al., 2006).

**TABLE 4:** Information of top ten most cited articles.

Paper	Title	DOI	Journal	Type	Total citations	Citations per year
FINKEL T, 2000, NATURE	Oxidants, oxidative stress and the biology of aging	10.1038/35041687	Nature	Review	6,605	287.1739
WOLPAW JR, 2002, CLIN NEUROPHYSIOL	Brain–computer interfaces for communication and control	10.1016/S1388-2457(02)00057-3	Clinical Neurophysiology	Review	4,672	222.4762
LIN MT, 2006, NATURE	Mitochondrial dysfunction and oxidative stress in neurodegenerative diseases	10.1038/nature05292	Nature	Review	4,146	243.8824
PACHER P, 2007, PHYSIOL REV	Nitric oxide and peroxynitrite in health and disease	10.1152/physrev.00029.2006	Physiological Reviews	Review	4062	253.875
NEUMANN M, 2006, SCIENCE	Ubiquitinated TDP-43 in frontotemporal lobar degeneration and amyotrophic lateral sclerosis	10.1126/science.1134108	Science	Article	4,034	237.2941
HAASS C, 2007, NAT REV MOL CELL BIO	Soluble protein oligomers in neurodegeneration: lessons from the Alzheimer’s amyloid β-peptide	10.1038/nrm2101	Nature Reviews Molecular Cell Biology	Review	3,583	223.9375
BROOKS BR, 2000, AMYOTROPH LATERAL SC-a-b-c	El Escorial revisited: Revised criteria for the diagnosis of amyotrophic lateral sclerosis	10.1080/146608200300079536	Amyotrophic Lateral Sclerosis and Other Motor Neuron Disorders	Article	3,474	151.0435
DANBOLT NC, 2001, PROG NEUROBIOL	Glutamate uptake	10.1016/S0301-0082(00)00067-8	Progress in neurobiology	Review	3,441	156.4091
DEJESUS-HERNANDEZ M, 2011, NEURON	Expanded GGGGCC hexanucleotide repeat in noncoding region of C9ORF72 causes chromosome 9p-Linked FTD and ALS	10.1016/j.neuron.2011.09.011	Neuron	Article	3,022	251.8333
BLOCK ML, 2007, NAT REV NEUROSCI	Microglia-mediated neurotoxicity: uncovering the molecular mechanisms	10.1038/nrn2038	Nature reviews neuroscience	Review	2,811	175.6875

### Journal analysis

A total of 3,038 journals had published ALS-related studies. We extracted the number of publications, total number of citations, number of citations per article, H index, JCR quartile, and impact factor. Table 5 lists the top 10 most productive journals.

**TABLE 5:** Overview of top 10 most productive journals in ALS research.

	Number of publications	Total citations	Citations per paper	H index	Quartile	IF
Amyotrophic Lateral Sclerosis and Frontotemporal Degeneration	1,542	39,408	25.5564	79	Q2	3.528
Plos One	556	19,314	34.7374	67	Q2	3.752
Muscle and Nerve	469	14,303	30.4968	56	Q2	3.852
Neurology	448	34,810	77.7009	105	Q1	11.800
Neurobiology of Aging	407	12,115	29.7666	55	Q2	5.133
Journal of the Neurological Sciences	399	13,124	32.8922 =	60	Q2	4.553
Acta Neuropathologica	381	26,963	70.7690 =	91	Q1	15.887
Human Molecular Genetics	339	21,436	63.2330 =	82	Q1	5.121
Journal of Neurochemistry	325	19,566	60.2031	82	Q2	5.546
Neurobiology of Disease	323	15,416	47.7276	68	Q1	7.046

As a specialty journal on ALS research, ALS *and Frontotemporal Degeneration* had published the highest number of ALS articles and was the most cited journal. First established as ALS *and Other Motor Neuron Disorders* in 2000, this journal had published 1,542 articles and received 39,408 citations. Its impact factor had increased from 0.848 to 3.528 in 2021, ranking 106/212 in “clinical neurology,” showing a tenacious growth potential. The journal with the highest H index was *Proceedings of the National Academy of Sciences of the United States of America*. Although not listed in Table 5, the journal with the highest number of citations per article was *Annual Review of Genetics*, with 2,302 citations on average.

### Keyword co-occurrence network

**FIGURE 3:**
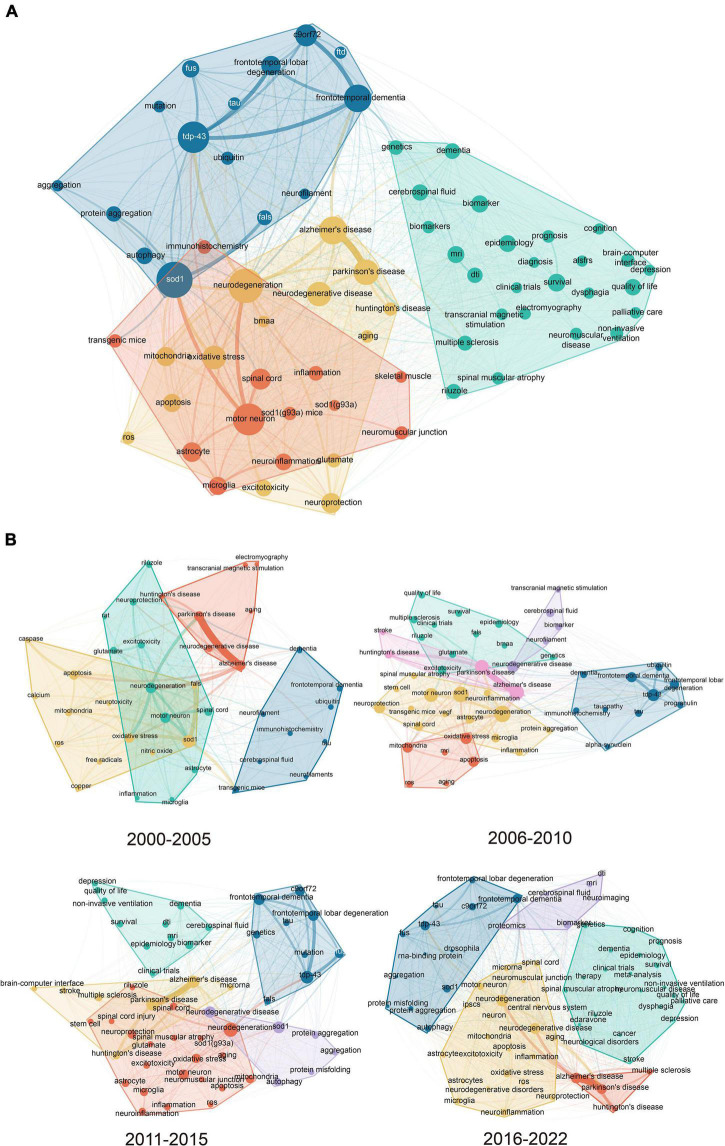
**(A)** Keyword co- occurrence network with clustering. **(B)** Subgroup analysis by time period in the keyword co-occurrence network (the circle size represents the frequency of occurrence; the color represents the cluster wherein the keyword belongs to; and the line thickness between two circles represents how two keywords co-occurred with each other in the publications).

A keyword co-occurrence network with a frequency greater than 100 was created, as shown in Figure 3A. The network presented a tightly knit cluster representing the strength of the linkage and major trends in research topics since 2000. The node size reflected the occurrence frequency of keywords, indicating the importance of keywords. The thickness of the line indicated the strength of the connection between two nodes, which was formed by the frequency of their co-occurrence. Four clusters were generated in the results, color-coded, and roughly grouped into the following topics.

The blue cluster in the upper left corner was mainly related to the molecular mechanism underlying the pathophysiology of ALS, including SOD1, C9orf72, TDP-43, and protein aggregation.

In the right corner, the green cluster reflected the clinical research content of ALS, including the diagnosis, symptoms, treatment, and prognosis. The keywords were as follows: genetics, dementia, cerebrospinal fluid, transcranial magnetic stimulation, MRI, biomarker, dysphagia, riluzole, and palliative care.

Two overlapping clusters were presented below the network. The red cluster reflected research directions related to motor neurons, such as in SOD1(G93A) mice, astrocytes, microglia, and neuroinflammation. The yellow cluster reflected diseases associated with neurodegeneration and their common mechanisms: Alzheimer’s disease, Parkinson’s disease, autophagy, oxidative stress, and aging.

Figure 3B presents the subgroup analysis by time period in the keyword co-occurrence network. The time span was divided into four periods: 2000–2005, 2006–2010, 2010–2015, and 2016–2022. Co-occurrence networks were generated using the same algorithm, and keywords were classified into clusters for each period. Owing to the update of the content, the meaning of the same color cluster was not consistent across different periods.

### Keyword dynamics

**FIGURE 4:**
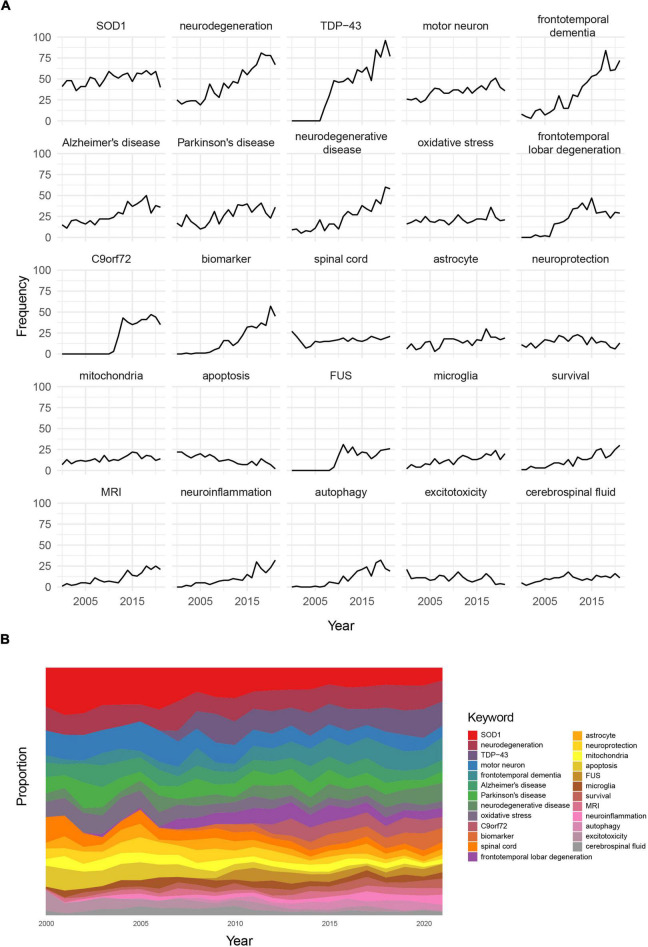
**(A)** Frequency dynamics for the top 25 most used keywords. **(B)** Proportion trends for the top 25 most used keywords (the color of the strip represents different keywords, and the width of the strip represents their proportion).

Generally, dynamic trends in keywords explain the popularity of a particular field. Figure 4A shows the absolute frequency of the top 25 most used keywords and their relative frequencies over the periods. The frequency of occurrence of most research topics, such as neurodegeneration, frontotemporal dementia, and Alzheimer’s disease, had increased. There had been explosive growth in the use of some keywords since their debuts, such as TDP-43, FUS, and C9orf72. The frequency of use of other keywords, such as neuroprotection, apoptosis, and excitotoxicity, decreased. Figure 4B shows the proportion dynamics for each keyword. Apart from the topics with decreasing frequency of use, topics, such as SOD1, also showed decreasing trends, despite their steady frequency.

## Discussion

Since Charcot first used the term “ALS” to describe the corresponding symptoms in 1874 (Goetz, 2000), this disease has been explored for a century and a half; however, its pathogenesis and treatment remain unclear to date. Bibliometric studies provide a broader perspective on many achievements in this process and a more profound understanding of the contributions of various countries, journals, and authors, as well as important research nodes and development trends.

A total of 96 countries and regions had contributed to ALS research. Compared with the description by Ram (2016), the descriptions by the top 10 countries were broadly similar, but with some changes: Italy had overtaken Japan in second place; China had moved from outside the top 10 to fifth place; and Australia had overtaken Spain. An output growth well above average was observed in other Asian and Middle Eastern countries, including India, South Korea, Iran, and Israel. Generally, the socioeconomic volume indicated by gross domestic product is closely related to research output. Countries with rapid economic growth or large economies are expected to invest more in research output (Shelton, 2008). Given the high rate of economic growth in these regions (World Bank, 2020), an increase in research output should be correlated with economic development.

As illustrated in Figure 1B, the cooperation network between countries and regions could be divided into three clusters among the top 50 countries contributing to ALS research. Collaboration with the United States caused the other countries to be included in the blue cluster, although they had scarce cooperation with each other in this cluster. Residents of most of these countries do not speak English as their native language, and these countries have a lower proportion of multiple country papers (M), indicating less participation in international research. The green cluster was mainly composed of European Union countries, whose scientific research cooperation was decentralized, and the cooperation curve showed network crossing. Belgium, Ireland, Switzerland, and Sweden had impressive MCP ratios. Although Turkey was also included in this cluster, it had an abnormally low MCP ratio. As a member of the European Union, Turkey is quite different from major European countries in terms of culture (Scherpereel, 2010; Gungor et al., 2012). This shows that in addition to political factors, language and culture have great contributions to the resistance to the development of international research.

The articles published in English journals by non-native English authors constituted a relatively small portion of the total number of publications in these countries. For instance, most of the scientific results in Russia were published in Russian-language journals (Kotsemir, 2012); their actual contribution to ALS research may be insufficient in our analysis, especially because non-English language articles were rarely cited in international articles (Man et al., 2004). Our restriction to English might have led to language- and information-screening bias, and we might have consequently underestimated the contribution of non-English-speaking countries.

Notably, the differences in the publication contribution between countries showed that the available knowledge on ALS is still heavily skewed toward Western countries, as in other scientific fields (Henrich et al., 2010). Therefore, our understanding of ALS may be lacking because of this systemic bias. Collaborative research combining different countries could help bridge the gaps in knowledge.

Using a co-occurrence analysis, we found a research focus in this field. The keywords, as the most important part of articles, were used to create a co-occurrence network map. As shown in Figure 3A, all keywords from 2000 to 2022 were classified into four clusters: molecular mechanism (blue), clinical research (green), cellular pathological change (red), and neurodegeneration (yellow). SOD1, TDP-43, frontotemporal dementia, and C9orf72 appeared to be pivotal in the molecular mechanisms of ALS. The content of clinical research was relatively scattered, and the main content focused on survival, biomarkers, diagnosis, and quality of life. Cellular pathological changes mainly occurred in the motor neurons, spinal cords, astrocytes, and microglia. Neurodegeneration research focused on Alzheimer’s disease, Parkinson’s disease, and oxidative stress.

The subgroup analysis of the co-occurrence networks demonstrated that since 2000, research on all aspects of ALS had made great progress, which mainly focused on clinical and molecular research. In terms of the molecular mechanism, the theme of each stage continued to be updated and expanded. From the initial SOD1 to TDP-43, C9orf72, and RNA-binding protein hypothesis in recent years, the research vision is constantly expanding. However, progress in clinical research is seriously out of sync. Treatment research proceeded slowly. Currently, therapeutic drugs are restricted to riluzole and edaravone, and their effects are far from satisfactory (Miller et al., 2012; Jaiswal, 2019). The main clinical research progress lies in the diagnosis (e.g., MRI, DTI, and biomarkers) and improvement of quality of life (e.g., palliative care, depression, and noninvasive ventilation) (Hobson and McDermott, 2016; Niedermeyer et al., 2019; Kassubek and Müller, 2020; Shi et al., 2021; Verde et al., 2021; Yang et al., 2021).

Herein, the top 25 topics were mainly related to the pathophysiological mechanism of ALS, in which there was also dynamic differentiation. Since their discovery in 2007 and 2011, TDP-43 and C9orf72 have attracted great attention, respectively (Neumann et al., 2006; DeJesus-Hernandez et al., 2011; Renton et al., 2011). Although the relative frequency of SOD1 gradually decreased, its absolute frequency remained stable. This phenomenon may be attributed to the evolution of SOD1 research topics, from loss-of-function and oxidative stress in the beginning to prion-like transmission mechanisms in recent years (Rotunno and Bosco, 2013; Silverman et al., 2016; Salvany et al., 2022). Some topics, such as excitotoxicity and apoptosis, have gradually attracted less attention. The process of excitotoxicity is relatively clear (Blasco et al., 2014; King et al., 2016), and the drug developed for this purpose is not ideal (Cheah et al., 2010; Miller et al., 2012), which may account for the reduction in research on excitotoxicity. The marginalization of apoptosis is mainly attributed to necroptosis as the mode of death of motor neurons (Morrice et al., 2017).

Based on the co-occurrence networks and keyword dynamics, we obtained future research directions for ALS. Molecular mechanism research may focus on C9orf72 and the possible unifying pathomechanism of ALS, while clinical research may concentrate on quality of life and biomarkers for prognosis and diagnosis.

Our study has some limitations. First, for the sake of the quality of the database and the credibility of the source, the data for our bibliometric analysis were limited to data from the articles retrieved from the WOS Core Collection database. Databases with broader coverage but less rigorous screening processes, such as Google Scholar and PubMed, were not considered. Therefore, our analysis results could only be used as a representative result for a particular database. Second, owing to the limitations of literature research, there is some systematic bias in the length-time effect: Earlier published articles will receive more citations, and earlier topics will occupy a larger proportion.

## Conclusion

In conclusion, the output of ALS research has steadily increased over the years. The United States and Western Europe are the leaders in this field. Nevertheless, the contribution of Asian countries, such as China and India, is increasing rapidly. The most productive and influential researchers are from English-speaking countries. The most cited document on ALS is that by Finkel and Holbrook. The most productive and cited journal is ALS *and Frontotemporal Degeneration*. Our current ALS research directions mainly focus on molecular mechanisms, clinical research, cellular pathological changes, and neurodegeneration. In recent years, studies have mainly focused on clinical research and molecular pathomechanisms. We hope that our analysis results will be helpful in future ALS research and publications.

## Data availability statement

The original contributions presented in this study are included in the article/supplementary material, further inquiries can be directed to the corresponding author/s.

## Author contributions

F-FB and KH supervised the project and did all the revisions. GS and JZ extracted the data and performed the analysis. GS drafted the manuscript. All authors contributed to the conception and design of the study, provided critical feedback on drafts, and approved the final manuscript.
